# Mini - social phobia inventory (mini-SPIN): psychometric properties and population based norms of the German version

**DOI:** 10.1186/s12888-017-1545-2

**Published:** 2017-11-25

**Authors:** Jörg Wiltink, Sören Kliem, Matthias Michal, Claudia Subic-Wrana, Iris Reiner, Manfred E. Beutel, Elmar Brähler, Rüdiger Zwerenz

**Affiliations:** 1 Department of Psychosomatic Medicine and Psychotherapy, University Medical Center of the Johannes Gutenberg University Mainz, Mainz, Germany; 2 Criminological Research Institute of Lower Saxony, Hannover, Germany; 3 Institute of Psychology, Technical University of Braunschweig, Braunschweig, Germany

**Keywords:** Social anxiety disorder, Mini social phobia inventory, Mini-SPIN, Reliability, Validity, Cut-off, Norms

## Abstract

**Background:**

A short screening for social anxiety disorder is useful in clinical and epidemiological contexts. However, the German version of the short form of the Social Phobia Inventory (mini-SPIN) has not been evaluated yet. Therefore, our aim was to determine reliability, validity and population based norms of the German mini-SPIN.

**Methods:**

The mini-SPIN was evaluated in a clinical (*N* = 1254) and in a representative community sample (*N* = 1274). Clinical diagnoses, the Patient Health Questionnaire depression (PHQ-9) and somatization modules (PHQ-15), the Generalized Anxiety Disorder Scale (GAD-7), the Liebowitz Social Anxiety Scale (LSAS), and the Short-Form-12 Health Survey (SF-12) were used in the clinical sample. In the community sample, participants filled out socio-demographic and health related questions and short versions of the PHQ (PHQ-2, GAD-2, panic item). Internal consistency, test-retest reliability, sensitivity to change, discriminant validity, and convergent validity were examined. Receiver operating characteristic curve analyses were performed to determine cut-off scores. Population based norms were computed from the community sample.

**Results:**

We found internal consistencies between 0.80 and 0.83. Test-retest correlation was Rho = 0.61; sensitivity to change was comparable to the LSAS. Correlations indicated good convergent and discriminant validity of the mini-SPIN. Strict measurement invariance can be assumed regarding age and gender. Receiver operating characteristic curve analysis suggested a cut-off of 6 or higher for a probable diagnosis of SAD.

**Conclusions:**

The German version of the mini-SPIN is a reliable and valid instrument. Its brevity makes it valuable for screening and assessing changes of social anxiety in clinical and epidemiological studies.

## Background

According to DSM-5 (Diagnostic and statistical manual of mental disorders, 5th edition) social anxiety disorder (SAD) is marked by fear of situations where the individual is exposed to scrutiny by others; this may include interaction, observation or performance situations. The fears will act in a way or show anxiety that will lead to being negatively evaluated [1]. Social situations almost always provoke anxiety and are avoided or endured with intense fear or anxiety. The fear/anxiety is out of proportion to actual threat. The fear/anxiety/avoidance has lasted 6 months, leads to significant distress or functional impairment, is not due to a medical condition/drug or another mental disorder and either unrelated to existing medical conditions [1].

The average 12-month prevalence of SAD in the German population is 2% [2, 3] and 7.4% in the US population [4]. Women are more likely than males to develop SAD; mean age of onset is between age 10 and 16.6 years [5, 6]. It is a chronic and disabling disorder often accompanied by comorbid depression, personality disorders, other anxiety disorders or substance abuse [5]. Keller indicates that only a minority of patients with SAD attain full remission within 8 years [7]. Mistaken as shyness, SAD is often not recognized and therefore untreated [5, 7]. Because SAD remains undiagnosed – even in psychosomatic outpatient and consultation-liaison services – valid screening instruments are urgently needed [8]. There are several valid questionnaires available assessing social anxiety (performance anxiety and/or anxiety in interactions); e.g. Liebowitz Social Anxiety Scale, LSAS [9–11], Social Phobia Scale, SPS [12], Social Interaction Anxiety Scale, SIAS [12]. SIAS and SPS each consists of 20 items, the LSAS consists of 24 items for the assessment of anxiety and 24 items for the assessment of avoidance. All of these instruments are relatively long and therefore not feasible in settings with the need of brief orientation on symptoms (e.g. in general practice).

Connor et al. [13] derived a short form with three items from the 17-item self-administered Social Phobia Inventory (SPIN, [14]; German version [15]).

Its three items are supposed to discriminate between individuals with generalized social anxiety disorder and controls: “Fear of embarrassment causes me to avoid doing things or speaking to people”, “I avoid activities in which I am the centre of attention”, and “Being embarrassed or looking stupid are among my worst fears”. The 5-point-Likert rating scale ranges from 0=“not at all” to 4 = “extremely”. Using a cut-off score of 6 (range 0–12), the English version of the mini-SPIN has demonstrated sensitivity of 89% and specificity of 90% for detecting generalized social anxiety disorder [13, 16, 17]; psychometric properties of several translations in other languages have been demonstrated: Finnish [18], Spanish [19], Portuguese [20, 21].

The 17-item SPIN has been translated into German and translated back by systematic techniques to ensure the original meaning of the items. It was translated into German by a team of clinical psychology researchers and translated back by a bilingual clinical psychologist. Finally, the back-translated version of the German SPIN was reviewed and consensually approved by a team [15]. The three items of the German version of the mini-SPIN are identical with the three corresponding items of the German translation of the 17-item SPIN.

While the psychometric properties of the 17-item SPIN have been assessed in an earlier community survey [22], reliability and validity of the German short form (mini-SPIN) are unknown.

Compared to the longer 17-item SPIN and other existing scales a very short form of the questionnaire with sufficient psychometric properties is particularly less time consuming during assessment, thus more cost-efficient and applicable in clinical (e.g. general practice) and scientific contexts (e.g. community surveys).

Therefore, the aim of this study was to evaluate the German translation of the mini-SPIN in a clinical and in a representative community sample regarding its a) reliability (internal consistency, test-retest reliability) and b) discriminant and convergent aspects of validity. Further, we wanted c) to determine cut-off scores for the detection of social anxiety, and d) to determine population based norms.

## Methods

### Study 1 (clinical sample)

#### Participants

A total of *N* = 1254 patients have been treated in the inpatient and day hospital units of the Department of Psychosomatic Medicine and Psychotherapy of the University Medical Center of the Johannes Gutenberg University, Mainz between August 2010 and March 2015. Data were routinely collected according to the German law of data protection (130a BDSG) and in accordance with the guidelines in the Declaration of Helsinki.

The mean age of patients was 38.5 (Standard Deviation, SD 13.2) ranging from 16 to 78 years. 61% were female. 61% lived in a partnership. 48% had at least high school education. About one half of the sample was employed, 7.8% were on pension, 19.5% were unemployed, and the others reported schooling, part-time work or being responsible for household. The majority of 94% of the patients held German nationality.

Most of the patients were diagnosed with a depressive disorder (81.3%), 28.3% with somatoform disorder, 21.8% with agoraphobia/panic disorder, 12.9% with generalized anxiety disorder, 9.6% with eating disorder, and 8.3% with social anxiety disorder. Furthermore, 17% of the patients were diagnosed with a personality disorder. Mean duration of the inpatient or day hospital treatment was 48 (SD 19) days.

### Measures

In this inpatient and day hospital sample, mental disorders were clinically assessed by psychotherapists according to ICD-10 (International Statistical Classification of diseases, 10th edition [23]). Diagnoses were approved by the senior physicians or psychologists in regular supervisions.

Patients are routinely assessed at the beginning and at the end of their treatment by several questionnaires including measures on anxiety, depression and quality of life.

Depression was measured by the Patient Health Questionnaire (PHQ-9 [24, 25]). Examples for items of the PHQ-9 are: “Little interest or pleasure in doing things?” or “Poor appetite or overeating.” (0 = “not at all”, 1 = “several days”, 2 = “over half the days”, and 3 = “nearly every day”). Psychometric qualities of the PHQ-9 are comparable to clinical interviews [26]. Internal consistency of the PHQ-9 was good (Cronbach’s alpha = 0.88) [27, 28]. In a meta-analysis with more than 5000 participants in a primary care setting including 17 validation studies Gilbody et al. (2007) found a sensitivity of 92% and a specificity of 80% for the detection of major depression (cut-off > = 10) [29].

Anxiety was screened with the GAD-7 (Generalized Anxiety Disorder Scale, GAD-7 [30, 31]); e.g. “Trouble relaxing” (0 = “not at all”, 1 = “several days”, 2 = “over half the days”, and 3 = “nearly every day”). Internal consistency of the GAD-7 can be rated as good (Cronbach alpha = 0.89) [32]. A sum score of 10 and more indicates generalized anxiety with a good sensitivity (89%) and specificity (82%) [31].

Somatic symptoms were assessed with the PHQ-15 of the Patient Health Questionnaire [27, 33]. The questionnaire contains the 15 most common complaints covering the main DSM-IV criteria for the diagnosis of somatization disorder. Examples for items are: “Stomach pain” or “Dizziness” (0 = “not bothered a lot”, 1 = “bothered a little”, 2 = “bothered a lot”). The internal consistency of the PHQ-15 was good (Cronbach alpha = 0.89) [33]. For the PHQ-15 van Ravesteijn et al. found a sensitivity of 78% and a specificity of 71% for the detection of a somatoform disorder [34].

The Liebowitz Social Anxiety Scale (LSAS [9, 10]) was used to assess intensity of fear in 24 social situations (e.g. “Participating in small groups – having a discussion with a few others”; 0 = “none”, 1 = “mild”, 2 = “moderate”, 3 = “severe” fear or anxiety) and their avoidance in this situation (0 = “never”, 1 = “occasionally”, 2 = “often”, 3 = “usually”) by self-report. The LSAS demonstrates good internal consistency for the total score (Cronbach alpha 0.96) [11]. Considering sensitivity and specificity Mennin et al. (2002) identified a cut-off score of 30 for the probable diagnosis of a social anxiety disorder and a cut-off score of 60 for a generalized social anxiety disorder [9].

Subjective quality of life was assessed with the German version of the Short-Form-12 Health Survey (SF-12) as a common, reliable and valid instrument for evaluating various aspects of health status. It examines two main components by eight health-related concepts: The ‘physical health component’ (PHC; e.g. “Pain interferes with normal work”; 1 = “extremely”, 2 = “quite a bit”, 3 = “moderately”, 4 = “a little bit”) consists of the subscales ‘physical functioning’, ‘role-physical’, ‘bodily pain’ and ‘general health’; the ‘mental health component’ (MHC; e.g. “Felt calm and peaceful”; 1 = “none of the time”, 2 = “a little of the time”, 3 = “some of the time”, 4 = “a good bit of the time”, 5 = “most of the time”) contains the subscales ‘mental health’, ‘role-emotional’, ‘social functioning’ and ‘vitality’. As unit of measurement the total sum for both scales is calculated [35]. The reliability of the SF-12 was judged as satisfactory to good [35].

### Study 2 (community sample)

#### Participants

A representative German community survey was conducted by the USUMA GmbH (Unabhängige Serviceeinrichtung für Umfragen, Methoden und Analysen; independent service for surveys, methods and analyses in market and social research), which is an institute for demographic research. The German law of data protection (§ 30a BDSG, Bundesdatenschutzgesetz) was regarded and written consent was obtained. Ethics were weighted to the interests of the public and individuals concerned following 1823 (BGB, Bundesgesetzbuch) of the Civil Code of Law and in accordance with the guidelines in the Declaration of Helsinki. All data were collected by the end of 2006. Data assessment was based on 129 sample areas which represented the different socioeconomic structures of Germany. Households were selected randomly. The members of the households fulfilling the predefined inclusion criteria were also selected by random procedure. Participants were included when German was the native language and when they were 14 or more years of age. Firstly, 2157 addresses were attempted following a random procedure; 2079 of the addresses were valid. Selected persons were tried to contact for three times. (for detailed description of the data collection cf. [36]).

This survey was independent from the 2002 survey assessing the psychometric properties of the German 17-item SPIN. 1287 persons between 14 and 90 years agreed to participate (61.9% of valid addresses). All participants were contacted by trained interviewers in their homes. Self-rating questionnaires were presented. Interviewers offered help in case of difficulties to understand single questions. 13 subjects did not complete the mini-SPIN validly. Therefore, 1274 participants were included into further analysis. The sample was representative for the German population in terms of age, gender, and education.

Mean age was 48.8 (SD 18.2) ranging from 14 to 90 years. 54.2% were female. 54% of the participants were married, and 61% lived in a partnership. A total of 88% had less than high school education. Household income was mostly (75%) higher than Euro 1250 per month. One third of the sample was employed, whereas 31% were on pension and 6% were unemployed. The majority of 97% held German nationality. A total of 19% were residents of the Eastern states of Germany.

### Measures

Participants filled out standardised self-report inventories and on socio-demographic (e.g. age, gender, income) and health related questions (e.g. weight, height, health behaviour, health care utilization; for detailed description of the assessment cf. [36]). In addition to the mini-SPIN, we used the German version of the Patient Health Questionnaire (PHQ) to assess generalized anxiety with the two screening items of the GAD-7 [30, 31, 37]: “Feeling nervous, anxious or on edge”, “Not being able to stop or control worrying”. (0=“not at all”, 1 = “several days”, 2 = “over half the days”, and 3 = “nearly every day”). The internal consistency of the two items was good (Cronbach alpha = 0.82) [31]. A sum score of 3 and more (range 0–6) indicates generalized anxiety with good sensitivity (86%) and specificity (83%) [31]. Panic was assessed with the screening question of the PHQ [38]: “In the last 4 weeks, have you had an anxiety attack – suddenly feeling panic or fear?”. Item sensitivity for detecting a panic disorder is very good (93%), with a moderate specificity of 78% [38].

Depression was measured using the two-item depression module of the PHQ [39]: “Little interest or pleasure in doing things”, “Feeling down, depressed, or hopeless” 0 = “not at all”, 1 = “several days”, 2 = “over half the days”, and 3 = “nearly every day”. The internal consistency of the PHQ-2 was good (Cronbach alpha = 0.83). For the detection of major depressive disorder, a cut-off score of three has a sensitivity of 87%, and a specificity of 78%. Sensitivity for the detection of any depressive disorder was 79%, specificity 86% [39].

### Statistical analyses

Means, standard deviations, skewness and kurtosis were calculated for each item of the mini-SPIN. Additionally, we determined the corrected item-scale correlation for each item and Cronbach’s alpha for the scale [28]. For the population based norms we used cumulated percentages of the sum score of the scale separately for age and gender. Because scores are not normally distributed (especially in the community sample) non-parametric analyses (Mann-Whitney tests, Spearman-Rho correlations) were performed.

To test sensitivity to change, we calculated pre- to post-intervention within group effect sizes (ES_pre-post_) for the mini-Spin total score and the LSAS total score using the clinical sample. We subsequently compared the relevant ES_pre-post_ for the two measures and checked for any significant differences. ES_pre-post_ were calculated by standardizing pre-post/pre-follow-up mean differences for each intervention group by the standard deviation (SD) of the difference.

To determine optimal cut-offs, sensitivity, and specificity we used receiver operating characteristic (ROC) curves. We applied three criteria for these analyses. The clinical diagnosis of social anxiety was used. Being aware of the relatively low number of diagnoses (8.3%, also see [8]) we additionally used internationally validated cut-offs of the LSAS as criteria [9]. A cut-off of 60 indicated a generalized social anxiety and a cut-off of 30 a social anxiety.

These statistical computations were done with SPSS Statistics 23.

Level of significance was defined by *p* < .05; larger effects (*p* < .01, *p* < .001) were reported additionally. We did not perform alpha-adjustment because of the exploratory nature of the analyses.

To examine the levels of measurement invariance, a multi-group confirmatory factor analysis was conducted (MGCFA) using these group variable: Group 1: males <35 years; Group 2: males 35 to 50 years; Group 3: males 51 to 64 years; Group 4: males >64 years; Group 5: females <35 years; Group 6: females 35 to 50 years; group 7: females 51 to 64 years; Group 8: females >64 years.

**Fig. 1:**
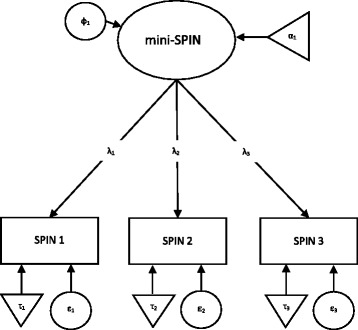
Explanation of the different models regarding measurement invariance analysis. Notes: Weak Invariance (Model0): λ1 = 1; λ_2__group A = … = λ_2__group H; λ_3__group A = … = λ_3_group H. Strong Invariance (Model1): α1_group A = 0; τ_1__group A = … = τ_1__group H; τ_2__group A = … = τ_2__group H; τ_3__group A = … = τ_3__group H; + weak invariance. Strict Invariance (Model2): Var (ε_1__group A) = … = Var (ε_1__group H); Var (ε_2__group A) = … = Var (ε_2__group H); Var (ε_3__group A) = … = Var (ε_3__group H); + weak and strong invariance. Strict Invariance (Model2b): Var (ε_1__group A) = … = Var (ε_1__group H) ≠ Var (ε_1__group C); Var (ε2_group A) = … = Var (ε2_group H); Var (ε3_group A) = … = Var (ε3_group H); + weak and strong invariance

In the case of partial measurement invariance (one or more model parameters identified, that were found to be variant across samples), we followed the recommendation of Byrne et al. (1989) to only conduct further invariance tests, when a minimum of two parameters per invariance test were found [40] (e.g., at least two factor loadings equivalent in metric invariance tests). If multivariate normality assumption was violated, we used the Satorra and Bentler’s (2001) scaling method [41]. We used a series of increasingly stringent model comparison steps to assess the factorial invariance of the mini-SPIN. First, weak invariance was tested. This is necessary for unbiased comparison of structural relationships (e.g., correlation coefficients, structural [path] coefficients) between latent constructs in different groups. Second, strong invariance was tested which allows the comparison of means of the latent construct between groups. Lastly, strict invariance was tested which allows unbiased decisions in screening processes that depend on the expression of a construct, resulting in different error rates (e.g., sensitivity, specificity) for different groups (see fig. 1 for further details of the different measurement models).

We used scaled CFI (comparative fit index) differences (ΔCFI) as well as scaled RMSEA (Root Mean Square Error of Approximation) differences (ΔRMSEA) to compare the difference stages of measurement invariance. As recommended by Chen (2007), a change of .010 in ΔCFIscaled, supplemented by a change of ΔRMSEAscaled = 0.015, was regarded as indicative of non-invariance [42].

To evaluate the goodness of fit of the relevant model in general, we follow the recommendation of Hu & Bentler (1999): A CFI > .900 was supposed for an *adequate* and a CFI > .950 for a *good* model fit [43]. Regarding the RMSEA a value of RMSEA < .050 were supposed for a close fit, values between.050 and .080 for a reasonably close fit, and values > .080 represent an unacceptable model fit. These analyses were conducted using the statistics software R (Version 3.2.5, [44]), with R Package lavaan [45].

## Results

### Study 1 (clinical sample)

#### Internal consistencies

Cronbach’s alpha of the three items for the clinical sample was 0.83. Table 1 displays the item characteristics and internal consistencies of the three items of the mini-SPIN. For better reading we reported scale means not sum scores (c.f. Table 1).

**Table 1:** Item and scale characteristics of the mini-SPIN

Original item (Connor et al. [14])	German translation (Stangier & Steffens [15])	Inpatient/ day hospital sample (*N* = 1082)					Representative community sample (*N* = 1274)				
		M	SD	Skew	Kurt	r_it_	M	SD	Skew	Kurt	r_it_
Fear of embarrassment causes me to avoid doing things or speaking to people.	Aus Angst vor Verlegenheit vermeide ich es, bestimmte Dinge zu tun oder Personen anzusprechen	1.62	1.26	0.30	−1.00	0.74	0.32	0.63	2.13	4.48	0.65
I avoid activities in which I am the centre of attention	Ich vermeide Aktivitäten, durch die ich im Mittelpunkt der Aufmerksamkeit stehe	1.93	1.29	0.03	−1.02	0.78	0.56	0.87	1.49	1.53	0.65
Being embarrassed or looking stupid are among my worst fears	Sich zu schämen oder dumm zu wirken, gehört zu meinen schlimmsten Ängsten	1.66	1.34	0.30	−1.10	0.79	0.32	0.66	2.46	6.79	0.69
Total Scale (mean)		1.74	1.12	0.18	−0.85	0.83^a^	0.40	0.61	1.86	3.62	0.80^a^

M = mean, SD = standard deviation, Skew = skewness, Kurt = kurtosis, r_it_ = corrected item scale correlation,^a^ Cronbach’s alpha; possible answers: 0 = “not at all” to 4 = “extremely”

### Test-retest reliability

The correlation between the mini-SPIN at beginning and the end of the treatment after a mean of 48 days of treatment was Rho = 0.61 (*p* < 0.001).

### Sensitivity to change

For the mini-Spin total score we found a pre- to post-intervention ES of 0.37 (95%-CI [0.31; 0.44]). For the LSAS we found a similar ES_pre-post_ = 0.33 (95%-CI [0.26; 0.40]). Thus, sensitivity to change seems comparable between both measures.

### Convergent and discriminant validity

In order to determine validity, the mini-SPIN was correlated with questionnaires covering similar (convergent validity) and divergent constructs (discriminant validity).

The mini-SPIN was closely related to social anxiety measured with the LSAS (Rho = 0.704, *p* < 0.001). It was also related to depression (PHQ-9, Rho = 0.485, *p* < 0.001), generalized anxiety (GAD-7, Rho = 0.455, *p* < 0.001) and somatization (PHQ-15, Rho = 0.266, *p* < 0.001). Lower scores in the mini-SPIN were associated with a higher mental health component regarding quality of life (MHC, Rho = −0.391, *p* < 0.001). There was no relation to the physical health component (PHC, Rho = −0.058, *p* = 0.070).

### Sensitivity and specificity

We determined sensitivity and specificity of different cut-offs of the mini-SPIN for different criteria: clinical diagnosis of a social anxiety disorder, generalized social anxiety based on LSAS >60, and social anxiety based on LSAS >30. For the criterion, clinical diagnosis of social anxiety disorder, we could analyze *N* = 1012 patients. A total of *N* = 87 (8.6%) were diagnosed with a social anxiety disorder. Mean age of them was 30 (SD = 9) years and *N* = 36 (41%) were female. For the two LSAS based criteria we could analyze *N* = 1007 patients. A total of *N* = 734 had a LSAS >30 (mean age 39 years, SD = 13; female *N* = 459, 63%); *N* = 405 had a LSAS >60 (mean age 38 years, SD = 13; female *N* = 252, 62%).

Results can be found in Table 2.

**Table 2:** Sensitivity and specificity of the mini-SPIN in a clinical sample

Cut-off	Clinical diagnosis of SAD (*N* = 87)^1^		LSAS > 60 ‘generalized social anxiety’ (*N* = 405)^2^		LSAS > 30 ‘social anxiety’ (*N* = 734)^2^	
	Sensitivity	specificity	sensitivity	specificity	sensitivity	specificity
1	1.000	.116	.993	.163	.960	.264
2	.989	.170	.993	.251	.947	.421
3	.966	.253	.978	.365	.896	.560
4	.920	.357	.938	.518	**.816**	**.718**
5	.874	.464	.881	.643	.714	.824
6	**.851**	**.568**	**.800**	**.756**	.604	.897
7	.690	.654	.694	.832	.495	.930
8	.552	.762	.541	.914	.354	.960
9	.448	.830	.415	.952	.257	.971
10	.310	.902	.262	.980	.155	.985
11	.218	.936	.180	.992	.105	.996
12	.092	.964	.096	.995	.056	.996

Bold type = cut-off with best balance regarding sensitivity and specificity; missing data: ^1^ *N* = 221, ^2^ *N* = 247

### Study 2 (community sample)

#### Internal consistencies

Cronbach’s alpha of the three items in the community sample was 0.80. Table 1 gives an overview.

### Correlates of the mini-SPIN

The mini-SPIN was unrelated to age (Rho = 0.015, *p* = 0.600). However, participants reaching the cut-off of at least 6 points were significantly younger (z = −2.03, *p* < .05). The frequency (days per week) of alcohol consumption (Rho = 0.000, *p* = 0.994) and the Body Mass Index (Rho = −0.048, *p* = 0.084) were unrelated to the mini-SPIN. It was significantly related to a bad subjective health status (Rho = 0.151, *p* < 0.001), depression (Rho = 0.374, p < 0.001) and generalized anxiety (Rho = 0.378, *p* < 0.001).

Female (compared to male) participants and participants with panic attacks reported higher scores in the mini-SPIN (z = 3.736, *p* < 0.001 resp. z = 8.470, *p* < 0.001).

### Factorial invariance

**Fig. 2:**
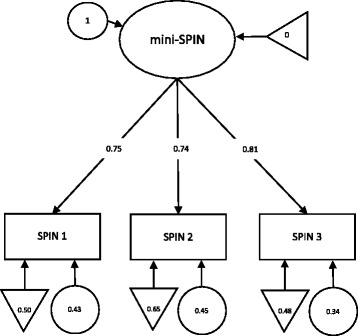
Factor loadings, intercepts, and residual variances for a unidimensional measurement model using the entire sample

A baseline model (Model 0), which simultaneously estimated all model parameters constraining all factor loadings to be invariant across aforementioned groups resulted in excellent model fit (CFIscaled = 1.0; RMSEAscaled = 0.000 [CI: 0.000, 0.000]). Strong invariance was examined by comparing Model 0 with Model 1 (see Table 3), which constrained all item intercepts to be invariant across groups. ΔCFI were below the cut-off recommended by Chen. Furthermore, the model fit was excellent (CFIscaled = 0.996; RMSEAscaled = 0.020 [CI: 0.000, 0.058]). Therefore, weak invariance can be assumed. Strict Invariance was examined by comparing Model 1 with Model 2a, which constrained all item residual variances to be invariant across groups, resulting in a considerable worsening of model fit (ΔCFI = −0.028; supplemented by ΔRMSEA = 0.021). Subsequently, one item residual variance in one group were freed, the resulting Model 2b exhibited an exactable difference in fit compared with Model 1 (ΔCFI = −0.007; ΔRMSEA = +0.004). Furthermore, the model fit was excellent (CFI = 0.989; RMSEA = 0.024 [0.000, 0.048]). Thus, strict invariance can be assumed for the mini-SPIN regarding age and gender. Figure 2 illustrates the factor loadings, factor intercepts, and item residual variances for a unidimensional measurement model using the entire sample.

**Table 3:** Measurement invariance of the mini-SPIN

		χ^2^_scaled_	df	CFI	ΔCFI	RMSEA	ΔRMSEA	Measurement Invariance Test^a^
Model 0	weak invariance	5.63	14	1.000	–	0.0	–	√
Model 1	strong invariance	25.65	27	.996	−.004	.020	+.020	√
Model 2a	strict invariance	61.00	48	.968	−.028	.041	+0.21	x
Model 2b	Strict invariance (partial)	51.40	47	.989	−.007	.024	+.004	√

df = degrees of freedom; CFI = Comparative Fit Index; ΔCFI = differences between models (0 and 1, 1 and 2a; 1 and 2b) regarding CFI; RMSEA = root mean square of approximation; ΔRMSEA = differences between models (0 and 1, 1 and 2a; 1 and 2b) regarding RMSEA^a^ **=** *ΔCFI* ≤ −*.010* supplemented by *ΔRMSEA ≥ .015* indicates non-invariance. √ marks invariance

### Population based norms

Table 4 provides a detailed illustration of the population based norms of the mini-SPIN. We display norms separately for gender (female, male) and age groups (<30, 31–40, 41–50, 51–60, 61–70, >70 years). Percentiles (cumulative percentages) are displayed for the sum scores of the scale.

**Table 4:** Population based norms of the mini-SPIN (representative community sample, *N* = 1274)

Cumulative %												
Score	female						male					
	<=30y	31-40y	41-50y	51-60y	61-70y	>70y	<=30y	31-40y	41-50y	51-60y	61-70y	>70y
	*N* = 122	*N* = 125	*N* = 120	*N* = 115	*N* = 127	*N* = 82	*N* = 119	*N* = 87	*N* = 92	*N* = 97	*N* = 115	*N* = 73
0	59,0	52,8	59,2	48,7	50,4	50,0	63,0	59,8	60,9	67,0	61,7	60,3
1	66,4	60,8	64,2	63,5	64,6	64,6	73,9	73,6	77,2	76,3	73,9	75,3
2	77,0	70,4	77,5	78,3	78,7	76,8	84,0	86,2	84,8	86,6	81,7	80,8
3	88,5	83,2	87,5	85,2	91,3	84,1	94,1	92,0	91,4	91,8	88,7	89,0
4	92,6	88,8	89,2	91,3	96,1	93,9	96,6	96,6	92,4	94,8	93,0	94,5
5	94,3	89,6	95,8	96,5	99,2	100	98,3	100	95,7	97,9	94,8	97,3
6	96,7	92,8	96,7	99,1	100	100	100	100	96,7	100	96,5	98,6
7	97,5	97,4	96,7	99,1	100	100	100	100	97,8	100	98,3	100
8	98,4	99,2	99,2	100	100	100	100	100	100	100	99,1	100
9	99,2	99,2	100	100	100	100	100	100	100	100	100	100
10	99,2	99,2	100	100	100	100	100	100	100	100	100	100
11	100	100	100	100	100	100	100	100	100	100	100	100
12	100	100	100	100	100	100	100	100	100	100	100	100

## Discussion

Our aim was to evaluate the German version of the three item short form of the Social Phobia Inventory (min-SPIN) in a clinical and in a representative community sample.

Taking into account the shortness of the scale we found good internal consistencies (Cronbach’s alpha 0.80 to 0.83). Our results are therefore in the range of comparable studies of the mini-SPIN in other languages (e.g. [16, 19, 46]. Thus, there is no need for a revision of the mini-SPIN including different items to enhance internal consistency, as Aderka et al. (2013) have suggested [47].

Test-retest reliability (Rho = 0.61) was somewhat lower than in comparable studies (e.g. [16]). However, we assessed the mini-SPIN before and after treatment in our clinical sample. As patients were treated in our inpatient or day hospital setting treatments did not differ significantly. The therapeutic orientation of the multimodal treatment setting was psychodynamic including cognitive-behavioral and psychoeducational elements, art therapy, body-oriented therapy, relaxation therapy and physical therapy. However, patients were diagnosed with a broad spectrum of mental disorders leading to a heterogeneous psychotherapeutic outcome especially regarding social anxiety, which can be expected to lower the association between test and retest by reducing social anxiety in a part of the sample. Regarding the sensitivity to change, we found comparable pre- to post-intervention effect sizes for the mini-SPIN total score and the LSAS total score. The effect sizes were only small [48]. This might be due to the fact that reducing social anxiety was not the primary goal of most of the inpatient or day hospital treatments.

In our clinical sample, we found evidence of a good construct validity of the mini-SPIN. It was strongly related with measures of the same construct (LSAS) as an aspect of convergent validity. Correlations of the mini-SPIN with scales assessing different symptoms (PHQ-9, GAD-7, PHQ-15) were also significant but somewhat lower. The mentally disabling character of social anxiety [5] is reflected by the positive correlation of the mini-SPIN with the mental health component (MHC). However, as expected, the mini-SPIN was unrelated to the physical health component (PHC). The lacking relation to PHC and the lower correlations to symptoms reflecting different disorders and MHC can be interpreted as an aspect of discriminant validity.

In our community study relations of the mini-SPIN to anxiety (GAD-2) and depression PHQ-2) were of medium height and comparable to the correlations found in the clinical sample. The positive relations between mini-SPIN and a bad subjective health status and female sex were plausible and compatible with findings of previous studies [5]. In contrast to other epidemiological studies in our sample the mini-SPIN was unrelated to age (e.g. [49]) in correlational analyses. This finding was caused by a small variance in this sample due to a majority (80%) of participants only reaching 2 points or less in the mini-SPIN. However, comparing participants above the defined criterion (mini-SPIN > = 6 points) with those below, patients suffering from social anxiety were significantly younger. Moreover, there is evidence from the literature, that social anxiety is related to alcohol abuse (e.g. [5]). In our study the frequency of alcohol consumption (average of 1.82 days per week, SD 2.04) was unrelated to the mini-SPIN. Probably the lack of relation between mini-SPIN and frequency of alcohol consumption is due to a) disparate reasons for the consumption (e.g. to cope inadequately with social anxiety or a sociable life style with frequent but moderate alcohol consumption and not abuse) or compared to other studies due to b) differing assessment of alcohol consumption (not taking into account quantity, frequency of and functional impairment following from alcohol consumption).

Furthermore, evidence of strict measurement invariance by sex and age and the associated possibility of unbiased comparison of means, correlation coefficients, path coefficients within SEM (Structural equation modeling) as well as the possibility of undistorted screening decisions between aforementioned groups, appear to be explicitly relevant.

To examine diagnostic accuracy of the mini-SPIN we assessed sensitivity and specificity for the three criteria (study 1). We confirmed a cut-off of 6 for the German version of the mini-SPIN (e.g. [13, 16, 17] to be best balanced regarding sensitivity and specificity for the clinical diagnosis of SAD and generalized social anxiety (LSAS > 60) as criteria (c.f. Table 2). For social anxiety determined by a lower cut-off (LSAS > 30) sensitivity and specificity were best balanced at a mini-SPIN cut-off of 4. Noteworthy, is the fact, that a sufficient sensitivity is accompanied by relatively low specificity in all criteria, especially for the clinical diagnosis. The reasons for the low specificity might be the relatively seldom (8.3%) clinical diagnosis of SAD (e.g. [8]) and the lack of a non-clinical comparison group.

Based on data of a large community sample (*N* = 1274) we were able to determine population based norms for different age groups and sex.

The strengths of our studies are the large sample sizes allowing determination of cut-offs and population based norms. Our results are a) somewhat limited by the lack of a non-clinical comparison group to assess diagnostic accuracy, and b) by the lack of a standardized clinical interview to ensure the diagnosis of SAD.

Further studies on the mini-SPIN should address diagnostic accuracy and especially include a non-clinical comparison group and standardized diagnostic of SAD.

Despite its limitations, the results of our two validation studies encourage the use of the German mini-SPIN in different settings. Its brevity, its easy interpretation and its reasonable psychometric properties make it suitable as a screening instrument in clinical (e.g. primary care) and also in study contexts (e.g. psychotherapy trials, epidemiological studies). It can also be used as an easy to apply follow-up measure in clinical studies or during inpatient and outpatient psychotherapy.

## Conclusions

The German version of the mini-SPIN is a reliable and valid instrument. Its brevity makes it valuable for screening and assessing changes of social anxiety in clinical and epidemiological studies.

## Abbreviations

BDSG: Bundesdatenschutzgesetz
BGB: Bundesgesetzbuch
CFI: Comparative fit index
DSM-5: Diagnostic and statistical manual of mental disorders, 5th edition
ES: Effect size
GAD-2: Generalized Anxiety Disorder Scale, short version
GAD-7: Generalized Anxiety Disorder Scale
ICD-10: International Statistical Classification of diseases, 10th edition
Kurt: kurtosis
LSAS: Liebowitz Social Anxiety Scale
M: Mean
MGCFA: Multi-group confirmatory factor analysis
MHC: Mental health component of the SF-12
Mini-SPIN: mini Social Phobia Inventory
PHC: physical health component of the SF-12
PHQ-15: Patient Health Questionnaire, somatization module
PHQ-2: Patient Health Questionnaire, depression module, short version
PHQ-9: Patient Health Questionnaire, depression module
RMSEA: Root Mean Square Error of Approximation
ROC: receiver operating characteristic
SAD: Social Anxiety Disorder
SD: Standard deviation
SEM: Structural equation modeling
SF-12: Short-Form-12 Health Survey
SIAS: Social Interaction Anxiety Scale
Skew: skewness
SPIN: Social Phobia Inventory
SPS: Social Phobia Scale
USUMA: Unabhängige Serviceeinrichtung für Umfragen, Methoden und Analysen; independent service for surveys, methods and analyses in market and social research

## References

[1] American Psychiatric Association. Diagnostic and statistical manual of mental disorders (5th ed.). Arlington, VA, American Psychiatric Publishing; 2013.

[2] Wittchen HU, Jacobi F (2001). Die Versorgungssituation psychischer Störungen in Deutschland. Eine klinisch-epidemiologische Abschätzung anhand des Bundes-Gesundheitssurveys 1998. Bundesgesundheitsbl Gesundheitsforsch Gesundheitsschutz.

[3] Fehm L, Beesdo K, Jacobi F, Fiedler A (2008). Social anxiety disorder above and below the diagnostic threshold: prevalence, comorbidity and impairment in the general population. Soc Psychiatry Psychiatr Epidemiol.

[4] Kessler RC, Petukhova M, Sampson NA, Zaslavsky AM, Wittchen HU (2012). Twelve-month and lifetime prevalence and lifetime morbid risk of anxiety and mood disorders in the United States. Int J Methods Psychiatr Res.

[5] Wittchen HU, Fehm L (2003). Epidemiology and natural course of social fears and social phobia. Acta Psychiatr Scand.

[6] Beesdo-Baum K, Knappe S, Fehm L, Höfler M, Lieb R, Hofmann SG, Wittchen HU (2012). The natural course of social anxiety disorder among adolescents and young adults. Acta Psychiatr Scand.

[7] Keller MB (2003). The lifelong course of social anxiety disorder: a clinical perspective. Acta Psychiatr Scand.

[8] Wiltink J, Haselbacher A, Knebel A, Tschan R, Zwerenz R, Michal M, Subic-Wrana C, Beutel ME (2010). Soziale Phobie – eine im psychosomatischen Ambulanz- und Konsildienst unterdiagnostizierte Angsterkrankung?. Psychother Psychosom Med Psychol.

[9] Mennin DS, Fresco DM, Heimberg RG, Schneier FR, Davies SO, Liebowitz MR (2002). Screening for social anxiety disorder in the clinical setting: using the Liebowitz social anxiety scale. J Anxiety Disord.

[10] Stangier U, Heidenreich T. Die Liebowitz Soziale Angst- Skala (LSAS). In Collegium Internationale Psychiatriae Scalarum (Hrsg.), Internationale Skalen für Psychiatrie. Weinheim, Beltz; 2004.

[11] Heimberg RG, Horner KJ, Juster HR, Safren SA, Brown EJ, Schneier FR, Liebowitz MR (1999). Psychometric properties of the Liebowitz social anxiety scale. Psychol Med.

[12] Mattick RP, Clarke JC (1998). Development and validation of measures of social phobia scrutiny fear and social interaction anxiety. Behav Res Ther.

[13] Connor KM, Kobak KA, Churchill LE, Katzelnick D, Davidson JR. Mini-SPIN: A brief screening assessment for generalized social anxiety disorder. Depress Anxiety 2001; 14:137–140. 10.1002/da.1055 11668666

[14] Connor KM, Davidson JRT, Churchill LE, Sherwood A, Foa E, Weisler RH (2000). Psychometric properties of the social phobia inventory (SPIN): new self-rating scale. Brit J Psychiatr.

[15] Stangier U, Steffens M. Social Phobia Inventory (SPIN) – Deutsche Fassung. Frankfurt am Main: Psychologisches Institut der Universität Frankfurt am Main; 2002.

[16] Seeley-Wait E, Abbott MJ, Rapee RM (2009). Psychometric properties of the mini-social phobia inventory. Prim Care Companion J Clin Psychiatr.

[17] Weeks JW, Spokas ME, Heimberg RG (2007). Psychometric evaluation of the mini-social phobia inventory (mini-SPIN) in a treatment-seeking sample. Depress Anxiety.

[18] Ranta K, Kaltiala-Heino R, Rantanen P, Marttunen M (2012). The mini-social phobia inventory: psychometric properties in an adolescent general population sample. Compr Psychiatry.

[19] Garcia-Lopez L, Moore HT (2015). Validation and diagnostic efficiency of the mini-SPIN in Spanish-speaking adolescents. PLoS One.

[20] Osório FL, Crippa JA, Loureiro SR (2007). A study of the discriminative validity of a screening tool (MINI-SPIN) for social anxiety disorder applied to Brazilian university students. Eur Psychiatr.

[21] Osório FL, Crippa JA, Loureiro SR (2010). Further study of the psychometric qualities of a brief screening tool for social phobia (MINI-SPIN) applied to clinical and nonclinical samples. Perspect Psychiatr Care.

[22] Sosic Z, Gieler U, Stangier U (2008). Screening for social phobia in medical in- and outpatients with the German version of the social phobia inventory (SPIN). J Anxiety Disord.

[23] Dilling H, Mombour W, Schmidt MH (1991). Internationale Klassifikation psychischer Störungen ICD 10 Kapitel V (F).

[24] Löwe B, Gräfe K, Zipfel S, Witte S, Loerch B, Herzog W (2004). Diagnosing ICD-10 depressive episodes: superior criterion validity of the patient health questionnaire. Psychother Psychosom.

[25] Kroenke K, Spitzer RL, Williams JB (2001). The PHQ-9: validity of a brief depression severity measure. J Gen Intern Med.

[26] Spitzer RL, Kroenke K, Williams JB (1999). Validation and utility of a self-report version of PRIME-MD: the PHQ primary care study. Primary care evaluation of mental disorders. Patient health questionnaire. JAMA.

[27] Gräfe K, Zipfel S, Herzog W, Löwe B (2004). Screening psychischer Störungen mit dem "Gesundheitsfragebogen für Patienten (PHQ-D)". Ergebnisse der deutschen Validierungsstudie. Diagnostica.

[28] Cronbach LJ (1951). Coefficient alpha and the internal structure of tests. Psychometrika.

[29] Gilbody S, Richards D, Brealey S, Hewitt C (2007). Screening for depression in medical settings with the patient health questionnaire (PHQ): a diagnostic meta-analysis. J Gen Intern Med.

[30] Spitzer RL, Kroenke K, Williams JB, Löwe B (2006). A brief measure for assessing generalized anxiety disorder: the GAD-7. Arch Intern Med.

[31] Kroenke K, Spitzer RL, Williams JB, Monahan PO, Löwe B (2007). Anxiety disorders in primary care: prevalence, impairment, comorbidity, and detection. Ann Intern Med.

[32] Löwe B, Decker O, Müller S, Brähler E, Schellberg D, Herzog W, Herzberg PY (2008). Validation and standardization of the generalized anxiety disorder screener (GAD-7) in the general population. Med Care.

[33] Kroenke K, Spitzer RL, Williams JB (2002). The PHQ-15: validity of a new measure for evaluating the severity of somatic symptoms. Psychosom Med.

[34] van Ravesteijn H, Wittkampf K, Lucassen P, van de Lisdonk E, van den Hoogen H, van Weert H, Huijser J, Schene A, van Weel C, Speckens A (2009). Detecting somatoform disorders in primary care with the PHQ-15. Ann Fam Med.

[35] Bullinger M, Kirchberger I (1998). Der SF-36 Fragebogen zum Gesundheitszustand.

[36] Wiltink J, Tschan R, Michal M, Subic-Wrana C, Eckhardt-Henn A, Dieterich M, Beutel ME (2009). Dizziness: anxiety, health care utilization and health behaviour. Results from a representative German community survey J Psychosom Res.

[37] Löwe B, Spitzer RL, Zipfel S, Herzog W (2002). PHQ-D Gesundheitsfragebogen für Patienten (German Version of the Patient Health Questionnaire).

[38] Löwe B, Grafe K, Zipfel S, Spitzer RL, Herrmann-Lingen C, Witte S, Herzog W (2003). Detecting panic disorder in medical and psychosomatic outpatients: comparative validation of the hospital anxiety and depression scale, the patient health questionnaire, a screening question, and physicians diagnosis. J Psychosom Res.

[39] Löwe B, Kroenke K, Grafe K (2005). Detecting and monitoring depression with a two-item questionnaire (PHQ-2). J Psychosom Res.

[40] Byrne BM, Shavelson RJ, Muthén B (1989). Testing for the equivalence of factor covariance and mean structures: the issue of partial measurement invariance. Psychol Bull.

[41] Satorra A, Bentler PMA (2001). Scaled difference chi-square test statistic for moment structure analysis. Psychometrika.

[42] Chen FF (2007). Sensitivity of goodness of fit indexes to lack of measurement invariance. Struct Equ Model.

[43] LT H, Bentler PM (1999). Cutoff criteria for fit indexes in covariance structure analysis: conventional criteria versus new alternatives. Struct Equ Model.

[44] Core Team R (2016). R: a language and environment for statistical computing.

[45] Rosseel Y (2012). Lavaan: an R package for structural equation modeling. J Stat Softw.

[46] Fogliati VJ, Terides MD, Gandy M, Staples LG, Johnston L, Karin E, Rapee RM, Titov N, Dear BF (2016). Psychometric properties of the mini-social phobia inventory (mini-SPIN) in a large online treatment-seeking sample. Cogn Behav Ther.

[47] Aderka IM, Pollack MH, Simon NM, Smits JA, Van Ameringen M, Stein MB, Hofmann SG (2013). Development of a brief version of the social phobia inventory using item response theory: the mini-SPIN-R. Behav Ther.

[48] Cohen J (1988). Statistical power analysis for the behavioral sciences.

[49] Jacobi F, Höfler M, Strehle J, Mack S, Gerschler A, Scholl L, Busch MA, Maske U, Hapke U, Gaebel W, Maier W, Wagner M, Zielasek J, Wittchen HU (2014). Mental disorders in the general population: study on the health of adults in Germany and the additional module mental health (DEGS1-MH). Nervenarzt.

[50] Landeskrankenhausgesetz (LKG) vom 28. November 1986. (GVBl. S. 342), zuletzt geändert durch Gesetz vom 19. Dezember 2014 (GVBl. S. 302), BS 2126–3. http://landesrecht.rlp.de/jportal/?quelle=jlink&query=KHG+RP&psml=bsrlpprod.psml Accessed 14 Nov 2017.

